# Optical Properties of 2D Micro- and Nanostructures of ZnO:K

**DOI:** 10.3390/ma15217733

**Published:** 2022-11-03

**Authors:** Rocío Ariza, Ana Urbieta, Javier Solis, Paloma Fernández

**Affiliations:** 1 Laser Processing Group, Institute of Optics (IO-CSIC), Serrano 121, 28006 Madrid, Spain; 2 Department of Materials Physics, Faculty of Physics, Complutense University of Madrid, 28040 Madrid, Spain

**Keywords:** ZnO, luminescence, defects, optical resonant modes

## Abstract

ZnO nano- and microstructures doped with K were grown by the Vapor–Solid method. Wires and needles are the main morphology observed, although some structures in the form of ribbons and triangular plates were also obtained. Besides these, ball-shaped structures which grow around a central wire were also detected. Raman and cathodoluminescence investigations suggest that variations in morphology, crystalline quality and luminescence emissions are related to the different lattice positions that K occupies depending on its concentration in the structures. When the amount is low, K ions mainly incorporate as interstitials (K_i_), whereas K occupies substitutional positions of Zn (K_Zn_) when the amount of K is increased. Electron Backscattered Diffraction shows that ribbons and triangular plates are oriented in the (0001) direction, which indicates that the growth of this type of morphologies is related to distortions introduced by the K_i_ since this position favors the growth in the (0001) plane. In the case of the ball-shaped structures, the compositional analysis and Raman spectra show that they consist of K_2_SO_4_. Finally, the capability of the elongated structures to act as waveguides and optical resonators was investigated. Due to the size of the K ion, practically double that of the Zn, and the different positions it can adopt within the ZnO lattice (K_i_ or K_Zn_), high distortions are introduced that compromise the resonators performance. Despite this, quality factor (Q) and fineness (F) show acceptable values (80 and 10 at 544 nm, respectively), although smaller than those reported for doping with smaller size alkali, such as Li.

## 1. Introduction

The properties of zinc oxide (ZnO) have been extensively investigated in recent years, specifically the influence of doping with different elements. Among the most advantageous properties of this material, its wide bandgap (3.37 eV) and exciton binding energy (60 meV at room temperature) are of particular interest for the development of applications within electronics and optoelectronics [1]. However, in this regard, one of the great limitations of ZnO is the difficulty in growing p-type ZnO due to self-compensation phenomena. To overcome this limitation, Park et al. [2] analyzed doping effects with elements of groups I and V in order to generate p-type conductivities. Their results show that potassium is the best candidate to achieve a stable p-type doping [3,4,5]. The incorporation of potassium in substitutional positions favors the formation of shallow defects and in turn facilitates the formation of Zn vacancies [2].

However, potassium shows a large difference in ionic radius with respect to Zn, and this is especially important since the reticular distortions introduced by dopants can affect the defect structure of the material and, hence, their electronic and optical properties. Huang et al. [6] studied the behavior and stability of group I elements as dopants at different positions in the ZnO lattice. In this study, it is observed that Li and Na show similar behavior; however, due to its greater ionic radius, drastic differences in the properties are achieved when K is used as a dopant.

Potassium-doped zinc oxide has been previously grown by sol–gel [4,7,8,9,10], electrochemical synthesis [11,12], rf cathodic [13,14] or calcination [15,16,17] methods. The degree of K incorporation depends on the method used, but in all cases, drastic changes in the behavior of the material have been observed. This allows improving the properties of the material for the selected application by controlling the amount of dopant, enabling its possible use in a variety of applications. In particular, ZnO:K can be used in optoelectronic devices where a p–n junction is needed, since a stable p-type conductivity can be achieved [14,18]. In addition, control of the conductivity opens the door to other applications such as Graetzel solar cells (DSSCs) [12], devices for water decomposition by non-photocatalytic processes [15], humidity sensors [19], spintronic devices [10,17,20] and photocatalytic processes [11]. The possibility of obtaining composites formed by ZnO/K_2_SO_4_ also increases the applicability of these structures as photocatalysts [9]

On the other hand, the fabrication of optical resonant cavities is of interest for their potential use as lasers [21,22,23,24], sensors [25], immunosensors [26] or optical filters [27,28,29], among others. In this regard, previously reported works on Li-doped ZnO elongated structures show the formation of Whispering Gallery Cavity Modes in the structures that act as resonators with high quality factors (ca. 670 at 459 nm) [30]. However, although the formation of resonant optical cavities in ZnO doped with alkaline ions (lithium and sodium) has been studied [25], no examples of the use of ZnO:K in waveguides or resonant cavities have been published to our knowledge.

In this work, micro- and nanostructures of ZnO doped with potassium were grown. A detailed characterization of the composition, crystalline quality and optical properties was carried out. The amount of potassium that is introduced into the ZnO lattice is one of the key parameters in the behavior of ZnO:K [4,8,10,20], so the growth of structures with three different percentages of K source of initial precursor was performed. The results show excellent optical properties that, along with the possibility of p-type doping, would allow a better integration of ZnO:K in different optoelectronic devices. Moreover, the potential application of ZnO:K structures as waveguides and resonant optical cavities with good quality factors has also been established.

## 2. Experimental

**Figure 1:**
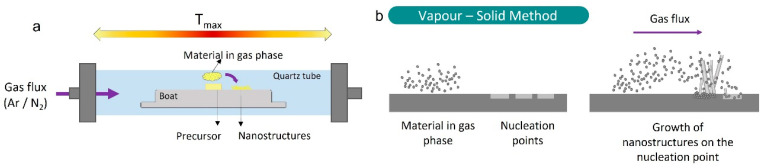
Scheme of the tubular furnace (**a**) and schematic drawing of the Vapor–Solid process (**b**).

The synthesis of K-doped ZnO structures was carried out using the Vapor–Solid (VS) method (Figure 1). As a Zn precursor, ZnS powder (Sigma-Aldrich, St. Louis, MO, USA, 99.99%) was used, while K_2_CO_3_ powder (Sigma-Aldrich, 99.0%) was selected as the K source. The initial content of K_2_CO_3_ was varied as 1%, 5% and 10% by weight. To simplify, the following notations are used: low (L) for 1%, medium (M) for 5% and high (H) for 10%. The powder mixtures were homogenized in an agate ball mill for 5 h and subsequently compacted under a 1T compressive load to form disk-shaped pellets. The samples were then placed on an alumina boat inside a quartz tube and annealed in a horizontal furnace at 900 °C for 10 h under a constant Ar flux of 1.7 L/min.

Morphological characterization was carried out by Secondary Electron (SE) mode in an FEI Inspect Scanning Electron Microscope (SEM, Lincoln, NE, USA). Cathodoluminscence (CL) and Energy Dispersive X-Ray (EDX) experiments were performed in a Leica 440 SEM (Wetzlar, Germany). CL images were recorded using a Hamamatsu R928 photomultiplier (Shizuoka ken, Japan) and CL spectra by means of a Hamamtsu PMA-11 charge coupled device; in both cases, an accelerating voltage between 15 and 20 keV was selected. For EDX detection, a Brucker AXS Quantax system was used (Billerica, MA, USA). Electron Backscattered Diffraction (EBSD) analysis was performed in an FEI Inspect SEM equipped with a Bruker Quantax e^−^ Flash 1000 EBSD system. µ-Raman and µ-photoluminescence (µ-PL) experiments were carried out in a Horiba Jobin Yvon Lab RAM HR800 confocal microscope. The 632.8 nm line of a He-Ne laser or the 325 nm line of a He-Cd laser were used as the excitation source. A 40× objective was used with the 325 nm excitation source, while a 100× objective was used for the Raman measurements. A special system in the confocal microscope enabled the detection of luminescence in a position of the sample separated from the excitation point [31]. Finally, XRD measurements were performed in a Philips diffractometer using Cu Kα radiation.

## 3. Results and Discussion

A high amount of nano- and microstructures were obtained after the thermal treatments. As a general observation, there are no significant variations in the types of morphologies present for each of the percentages of the precursors used. However, there are notable differences in the proportion in which each type of morphology occurs. The effect of dopants on the dominant morphology has been previously observed and attributed to two main factors: either changes in surface energy of the different faces or changes in the slip systems associated with stresses, both mechanisms rendering a different preferential growth direction [32,33]. Due to the temperature gradient that appears in the horizontal furnace, most of the structures grow onto the alumina boat, although some of them, in a much smaller quantity, also grow on the pellet surface.

**Figure 2:**
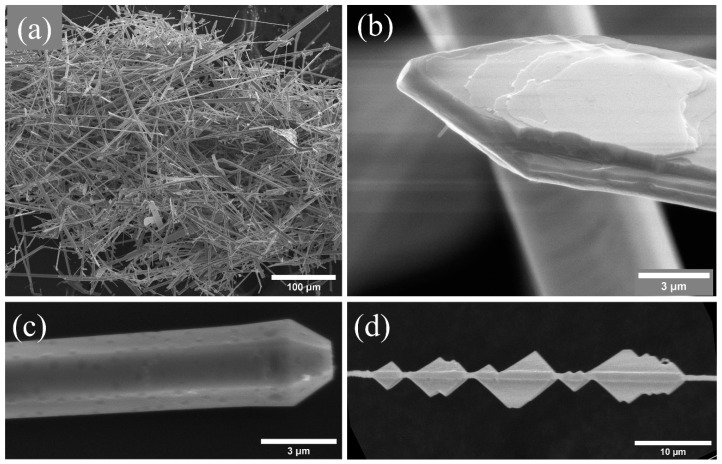
(**a**) General view of the morphologies of the structures grown, (**b**) detail of a ribbon with arrow ends, (**c**) needles with hexagonal cross-section and (**d**) central needle with triangle-shaped secondary growths.

To investigate the morphology of the grown structures, they were deposited on a silicon substrate or on graphite tape for analysis. In general, a high density of elongated structures in the form of wires and ribbons is obtained in all the samples, as shown in Figure 2a. Both morphologies have been previously observed for potassium-doped zinc oxide [11,15,16,17,18,19]. After a detailed analysis of the samples, three typical structures can be identified. Let us first focus on the ribbons, with widths between 10 and 20 μm, thicknesses of 2–3 μm and variable length, in the order of hundreds of microns. In particular, in Figure 2b, a detail of a ribbon with an arrowed end is shown. In some cases, as in the present example, their surface shows a series of steps that can be associated with intermediate phases of growth. The second type of structure is a needle with a hexagonal cross-section (Figure 2c). Such structures have an average diameter of 3 μm and a length of around 80–100 μm. This morphology is most likely to be observed, and it has also been reported in previous works for ZnO:K [11,12], indicating that K is incorporated as a dopant in these structures. Finally, structures with a central needle of diameter of about 700 nm and secondary growth in the form of triangular plates were obtained (Figure 2d). The triangles are about 2.5 μm in height. Several authors [15,17,34] have shown the appearance of plates with marked angles when doping with potassium, with a clear similarity to our structures. As already mentioned, potassium can occupy different positions within the ZnO lattice. The interstitial position (K_i_) is more likely to occur since it is the most energetically favorable. However, theoretical studies indicate that the diffusivity of K_i_ atoms is high and that they diffuse anisotropically, preferentially in the plane perpendicular to the c-axis of the hexagonal structure of ZnO [6]. This fact would explain the formation of plates and triangles, since this form of diffusion would facilitate growth in the plane (0001).

These observations allow us to infer the growth mechanism. As mentioned, there are no significant variations in the types of morphologies present for each of the percentages of the precursors. However, the proportion in which each type of morphology occurs drastically changes with K initial content. In L samples, the predominant morphologies are elongated structures such as needles or wires. On the other hand, for M and H samples, the number of plates increases significantly. This suggests that in the case of the low samples, the incorporation of K should be very low, while in the other two samples, dopant ions may be taking different positions in the lattice, inducing changes in the morphology.

**Figure 3:**
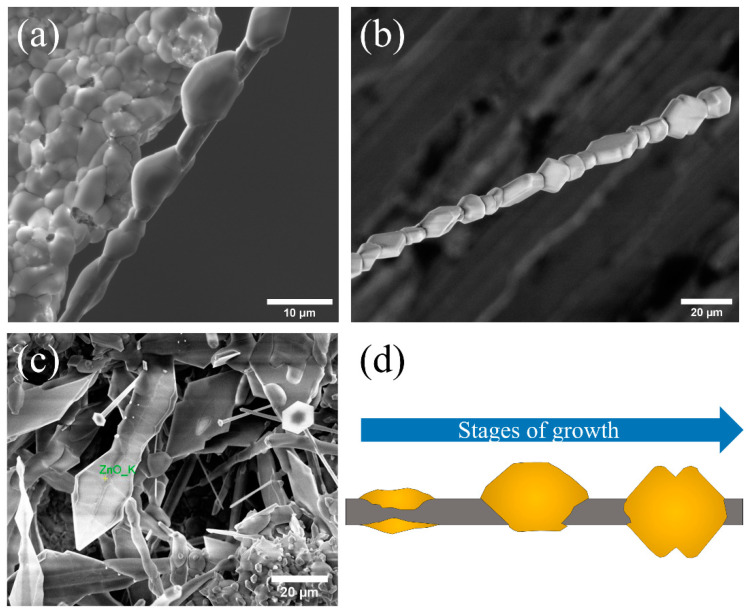
Characteristic morphologies observed on the surface of a pellet. (**a**) Quasi-periodic morphology that grows from a central thread. (**b**) Central thread coated with faceted structures. The orientation of each structure rotates 90° periodically (approximately every 30 μm). (**c**) Triangular structures. (**d**) Outline of how the growth of structures in (**a**,**b**) could occur.

As previously mentioned, although the growth is not abundant, the formation of some structures on the pellet has also been observed. In this case, their morphology is characteristic and consists of a series of small agglomerates that surround a central wire (Figure 3). This type of structure is not observed when the growth takes place on the alumina boat. Structures of this type can be found at different stages of growth, which also gives clues about the mechanism of formation. Figure 3a shows the initial stage of it. The image shows that a central wire with a diameter of about 4 μm is formed and the formation of small balls around it, with a diameter of ~10 μm, is initiated. As the heat treatment progresses, these balls appear faceted, showing slightly rounded edges (Figure 3b). In this structure, a certain periodicity can be observed in the orientation of each of the “necklace beads”. Approximately every 30 μm, the orientation rotates 90°. This type of growth from a central thread may be guided by the presence of a screw dislocation (Eshelby twist) along the main growth axis [35]. Finally, under the right conditions, the growth of triangular structures on the pellet with an approximate width of 20 μm can be also found, such as those shown in Figure 3c. Due to its similarity with the morphology and formation process previously found in structures of ZnO:Li grown by the same method [36], its chemical composition is expected to be that of a sulfate compound. Vogels et al. [37] report the theoretical morphologies for K_2_SO_4_ structures, with a result similar to the one observed here, as shown in Figure 3a,b. However, Figure 3c presents a slightly different morphology. Its origin is discussed later.

XRD patterns performed on the grown structures do not show phases different from ZnO wurtzite; however, a slight variation on the c parameter associated with the incorporation of K into the Zn lattice is observed. It changes from 5.2066 Å in the pure reference sample to 5.2078 Å in the L sample and 5.2080 Å in the M sample.

**Figure 4:**
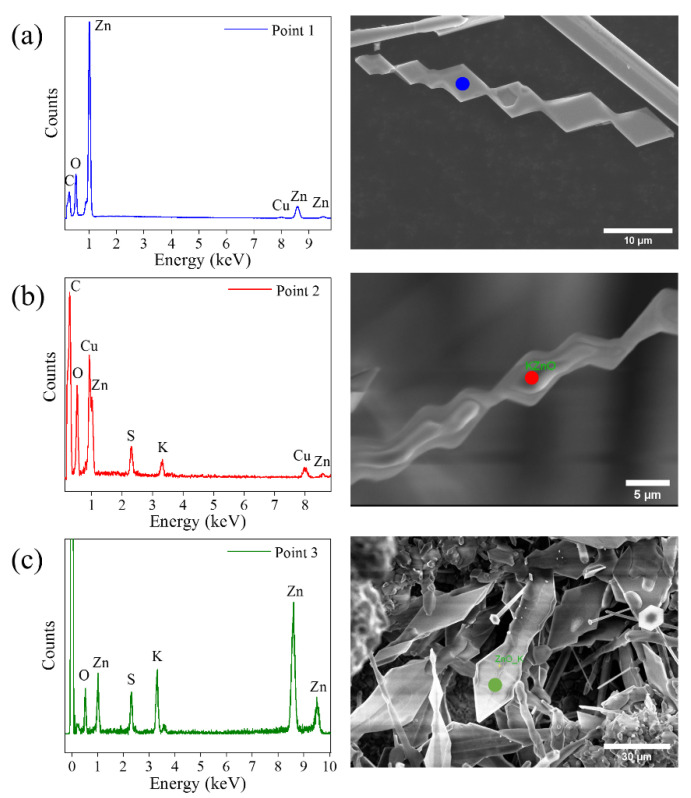
X-ray microanalysis on triangle-shaped structures. EDX spectrum taken on (**a**) a triangular structure without any agglomerate on the surface (blue point), (**b**) a triangular structure on which a second material has begun to grow (red point) and (**c**) a triangular structure (also shown in Figure 3c) with a slightly different morphology than those analyzed in (**a**,**b**) (green point).

The compositional analysis of the structures in the samples L-M-H was carried out by X-ray microanalysis (EDX). The presence of Zn and O was correctly identified. However, no presence of potassium was detected. This would indicate that the amount of K that can be accommodated into the ZnO lattice is below the detection limit of the technique. EDX spectra performed on triangular structures are shown in Figure 4. Measurements were made on several triangular structures at different stages of growth, where diamond-shaped plates along the axis can be observed. This morphology seems to derive from the joining of two triangles on the opposite sides of the wire, as observed in Figure 3b. Figure 4a shows these types of growths with a completely clean surface. The EDX spectrum for this structure detects Zn and O signals without measurable traces of K. In the second case (Figure 4b), the composition measured on another triangular structure, where the beginning of the deposit of a second material on the lateral faces is appreciated, was analyzed. The spectrum of this structure at the indicated point shows signals of O, Zn, S and K. This composition is consistent with the hypothesis that the material forming on the triangles is potassium sulfate.

Finally, the measurement shown in Figure 4c was performed on a wide structure with triangular peaks. The morphology differs slightly from the triangles observed in the first case, showing their lateral faces somewhat rounded. This structure could be the final phase of the coating of the triangles. The X-ray spectrum detects content in Zn, O, S and K. This result again supports the coating by K_2_SO_4_ while the signal of Zn and O would come from inside the structure. Gui-Yang et al. [6] show that when potassium is incorporated interstitially, it is able to diffuse quickly in the plane (0001), as explained above. However, depending on the amount of dopant that is introduced into the lattice, the solubility limit could be exceeded, and potassium would begin to diffuse out of the structure. At these dopant levels and for the morphologies observed, the quantifications are not accurate, but the K/O ratio increases by a factor close to 10, which is indicative of the different degrees of K incorporation. Looking at how the growth evolves in Figure 4, this excess of potassium in triangular structures could initiate the formation of potassium sulfate.

**Figure 5:**
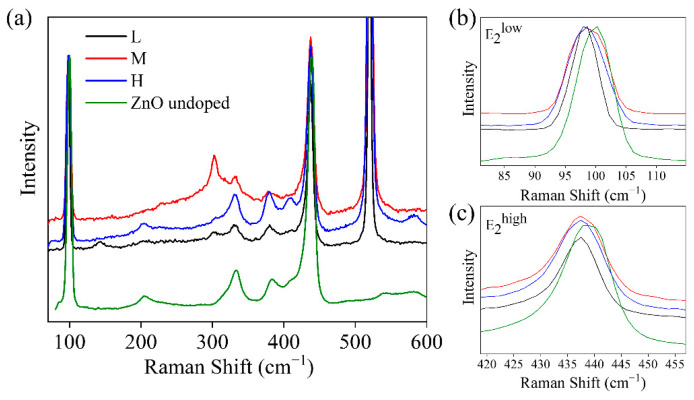
(**a**) Raman spectra of K-doped ZnO samples and undoped ZnO reference normalized to the peak of 98 cm^−1^. (**b**,**c**) Details of the peaks E_2_^low^ and E_2_^high^, respectively. A small displacement is appreciated with respect to the reference.

Raman spectroscopy provides information on crystalline quality and composition, allowing the behavior of dopants in the lattice to be analyzed. The measurements performed in the different types of samples are shown in Figure 5. The spectrum from pure ZnO is also shown for comparison. All the observed Raman modes match ZnO in the wurtzite phase [38], with two intense peaks observed at 98 cm^−1^ and 437 cm^−1^ that correspond to the E_2_ symmetries of the ZnO. No bands associated with second phases are observed in the spectra. Analyzing in detail the first of these peaks at 98 cm^−1^ (Figure 5b), a small displacement of 2–3 cm^−1^ with respect to pure ZnO is observed. The introduction of substitutional atoms can modify the spectrum of lattice vibrations, leading to a displacement of the Raman peaks. Potassium has an ionic radius (138 pm) approximately twice as much as that of Zn (74 pm), so the observed displacement could be due to the introduction of K into the ZnO lattice. The stress produced by this substitution can also cause these peaks to widen due to the loss of crystallinity. When calculating the full-width at half maximum (FWHM) of this peak for all samples (Table 1), a small widening is observed with respect to the reference. This broadening is also observed at the peak of 437 cm^−1^. In both cases, the M sample shows the greatest differences with respect to the pure ZnO, which could indicate either that this percentage would be maximizing the incorporation of K in the lattice or that potassium is being incorporated in different positions depending on the amount of doping.

This would be consistent with the fact, already mentioned, that alkaline elements can occupy different positions within the ZnO lattice and vary their position depending on the amount of dopant introduced [4,8,10,17,20]. The incorporation of potassium is energetically more favorable as interstitial than as Zn-substitutional. Unlike Li, when K is placed interstitially, it occupies a non-symmetrical position [6]. Kim et al. [8] suggest that K_i_ tends to move towards the vicinity of oxygen vacancies. This causes the crystallinity of the structures to improve, since potassium balances the distortion in the lattice caused by oxygen vacancies (V_O_), as has been reported experimentally [4,8,10]. The presence of K_i_ has also been observed to reduce the energy needed to displace a Zn atom from its equilibrium position, creating a zinc vacancy. Nevertheless, the diffusion of zinc is energetically expensive, and consequently, the migration of K from interstitial to substitutional Zn positions is not favored [6].

**Figure 6:**
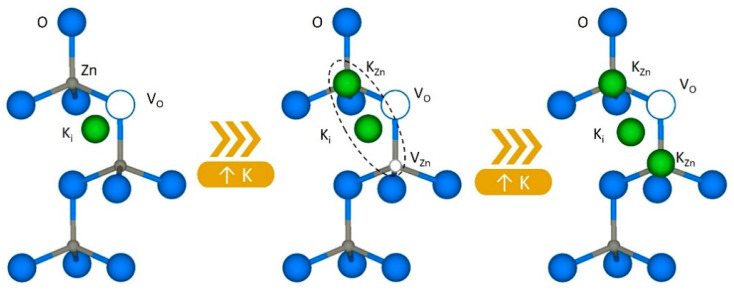
Schematic drawing of the changes occurred in the defect structure as a consequence of the incorporation of K^+^ into the Zn lattice.

However, when the amount of potassium incorporated increases above a threshold value (2 to 10 at %, depending on the growth method used in the literature), potassium begins to incorporate as Zn substitutional. The K_Zn_ position is stable, and diffusion may occur by exchange with V_Zn_ [6]. This has direct consequences on the structure of defects, as seen in the luminescence measurements, but also in the crystal structure. A reduction in crystallinity has been reported in the literature once this doping threshold is exceeded [4,7,8,17,19,20]. Kim et al. [8] report the formation of K-O bonds by XPS measurements when it exceeds 2% of K in the samples. K-O bonds are longer than Zn-O; hence, the substitution of Zn for K induces a noticeable distortion in the lattice that decreases crystallinity. This distortion has been detected by other authors via XRD [10,17], where they observe a sudden loss of crystallinity and an increase in the c parameter more noticeable than for the a parameter in the same percentage of incorporation of K. These authors argue that the incorporation of K_Zn_ occurs mostly in the c-axis. This is schematically drawn in Figure 6.

This last interpretation is consistent with our results of Raman spectroscopy and supports the idea that as the incorporation of K increases, its predominant position changes. For the L sample, we find the slightest displacement and broadening of the modes, while for the samples M-H, with higher K content, the distortion of the spectra is greater. The changes observed in intensities also agree with this assumption. As the amount of dopant increases, the intensity is gradually reduced, indicating a loss of crystallinity. In our case, however, there is no significant improvement in crystallinity before reaching the threshold of substitutional incorporation, as reported in the literature, which would indicate that the interaction with oxygen vacancies is slightly different.

**Figure 7:**
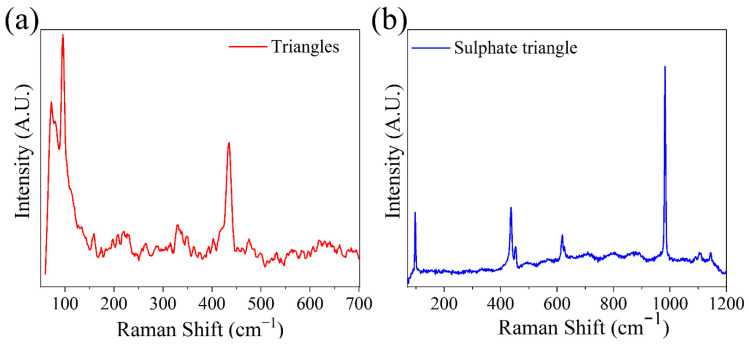
Raman spectra of triangle-shaped structures. Measurements on (**a**) a structure of uncoated triangles and (**b**) with growth of this second material.

µ-Raman spectroscopy measurements were additionally performed on the triangular structures. As mentioned regarding Figure 4, the triangular structures grow initially with a composition of Zn and O, and then begin to be covered with a second material containing S and K. Raman spectra were performed for triangular structures at both steps of growth, i.e., in clean structures (Figure 4a) and in structures fully covered with the second phase (Figure 4c). The results are shown in Figure 7. The signal collected on the clean triangles (Figure 7a) has a low intensity, but it is possible to differentiate the E_2_^low^ peaks at 96 cm^−1^ and E_2_^high^ at 434 cm^−1^. These peaks suffer a large displacement with respect to the positions previously observed. The crystallinity thus seems to be affected in these structures, which would coincide with a high incorporation of the dopant. This high rate of potassium accommodation in the lattice would explain the observed morphology. In turn, it would facilitate the initiation of potassium segregation and the subsequent formation of potassium sulfate. The spectrum obtained on these structures also shows two peaks at 72 and 80 cm^−1^. Measurements made by Ananthanarayanan et al. [39] on double sulfate K_2_Zn(SO_4_)_2_6H_2_O show Raman peaks at 65, 77, 90 and 100 cm^−1^ that could explain the modes detected at low frequencies in our experiments. However, the main vibrational modes of the sulfate anion have not been detected, so the possible formation of this compound has been ruled out.

In the case of fully coated triangles, a combination between the vibrational modes of ZnO and sulfate anion (SO_4_^2−^) is observed. Two peaks centered at 97 and 437 cm^−1^ associated with ZnO are observed. However, the dominant peaks are found at 454, 619, 626, 982, 1090, 1103 and 1145 cm^−1^ (Table 2). These peaks are consistent with those reported in the literature for potassium sulfate [40].

This result confirms that the material formed at the early state of the coating is potassium sulfate, by similarity with what was observed in the ZnO:Li structures [36]. The structures are ZnO triangles that have been completely coated by potassium sulfate, and there is neither co-doping of K and S in ZnO nor formation of a potassium–zinc double sulfate. In turn, since the ZnO modes were still detected, we can assume that the thickness of the coating is small, allowing us to obtain a signal from inside the structure. Hence, the ball structures form a core–shell composite of ZnO (inner thread)/K_2_SO_4_ (balls).

To further investigate the incorporation of K into the ZnO lattice, Electron Backscattered Diffraction experiments were performed on the grown structures.

**Figure 8:**
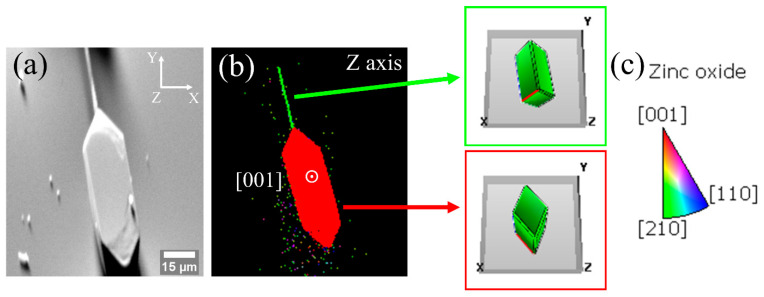
EBSD analysis. (**a**) Image of secondary electrons where the directions of the detector axes are fixed in the upper right corner. (**b**) EBSD map taken along Z axis. (**c**) Legend of the color scale used in EBSD maps. The colors indicate the proximity to the crystallographic directions indicated in (**b**).

Figure 8 shows EBSD measurements on a structure of the M sample where an elongated needle attached by one of its edges to a hexagonal plate is appreciated. Figure 8a is a secondary electron image of the structure on which the analysis has been performed. In this image, the orientation of the detector is indicated by the X, Y and Z axes in the upper right corner. The orientation map in the Z axis is shown in Figure 8b. The needle has an orientation slightly rotated between the directions [210] and [001], while the plate is close to [001]. To facilitate the visualization of how the crystallographic directions are oriented, Figure 8b includes the position of the unit cell for each part of the structure. The incorporation of potassium as K_i_ would favor the growth in the plane (0001) of the ZnO [6,15,17], forming hexagonal or triangular plates, while when incorporated as K_Zn_, substitutional Zn positions on the c-axis are more likely [10,17]. Our results suggest that K dopants are incorporated as interstitial in the plates and substitutional in the needle, reinforcing the assumption that the amount of K controls the type of morphology obtained.

**Figure 9:**
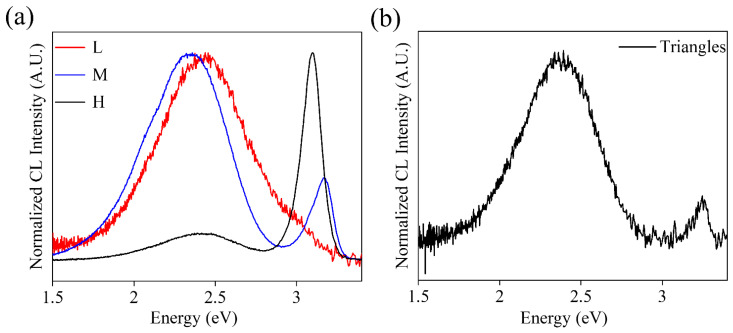
(**a**) CL spectra of elongated structures of L-M-H samples. (**b**) CL spectrum on a structure of triangles.

Cathodoluminescence (CL) experiments were also performed to study the luminescence properties of the grown structures. The normalized CL spectra of the K-doped ZnO samples are shown in Figure 9. The luminescence intensity varies greatly with the percentage of K precursors used. The lowest intensity corresponds to the lower sample (lower K content), while the M sample (medium K content) shows the highest intensity. This agrees with previously reported results which show an increase in luminescent intensity by increasing the potassium content up to an intermediate percentage of dopant. When this limit is exceeded, the intensity tends to suffer a slight decrease [4,6,7,10,17]. The spectra from the L sample show a single band in the visible range centered at 2.44 eV. As the potassium content increases (M sample), this band shifts towards 2.35 eV, and a small shoulder is observed at 2.10 eV. In turn, a new band in the UV range also appears at 3.17 eV. This band is not symmetric, indicating the presence of weak additional contributions in this energy range (2.92–3.17 eV). Finally, for the H sample, both the visible and UV bands are observed at 2.44 and 3.09 eV, respectively. In addition, the relative intensity of the visible band drastically decreases compared to the UV band. The visible band in the ZnO is usually attributed to the presence of deep level defects, where oxygen vacancies (V_O_) have an important contribution. Potassium doping has been shown to reduce the energy of oxygen vacancy formation [2,14,19,41], which would lead to an increase in the intensity of this band. On the other hand, contributions in the UV are associated with the band edge and the presence of shallow level defects. The M sample, as described, presents some asymmetry in the UV band due to weak contributions in the blue range. These contributions are associated with emissions related to V_Zn_ [42] and K_Zn_ [34], so a rise in the intensity of these bands is expected by increasing the amount of potassium incorporated [3,4,17,43].

The emission of the M sample undergoes a considerable shift with respect to that of the other two samples, which would indicate that potassium is acting differently, affecting the behavior of the defect structure. It is common in these percentages of doping the appearance of Burstein–Moss and/or band renormalization phenomena, which would also explain these differences. As mentioned above, Raman results also show a particular behavior for this sample.

In turn, CL spectra were also obtained for the triangles (Figure 9b); a dominant emission in the visible band centered at 2.38 eV and a less intense UV band at 3.25 eV are observed in this case. Both emissions shift slightly for these triangular structures. CL results may also vary depending on the orientation of the face being measured. Therefore, the observed displacements would indicate that the incorporation of the dopant is occurring in different positions and favoring some faces of the crystal structure over others, as EBSD experiments suggest, which also agrees with the observed variations in morphology.

**Figure 10:**
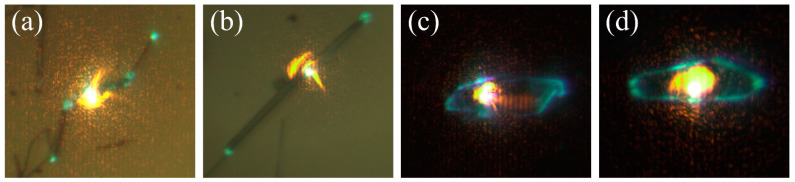
Optical microscopy images show the guidance of light that occurs within the structures, either in elongated structures (**a**,**b**) or in plates (**c**,**d**). The orange dot is the excitation laser (325 nm), while the blue-green light points correspond to the emission generated by photoluminescence that has been guided to the extremes.

µ-PL experiments have been carried out to investigate the optical behavior of the grown structures of ZnO:K, which have favorable light-guiding properties. By exciting a structure with the UV laser in its center, the light generated by photoluminescence is guided to the ends or edges of the structure. Figure 10 shows optical images of some illustrative examples of this guiding phenomenon. No losses are observed along the wires (a,b) nor across the plates (c,d), and only at the edges does light escape from the structure.

**Figure 11:**
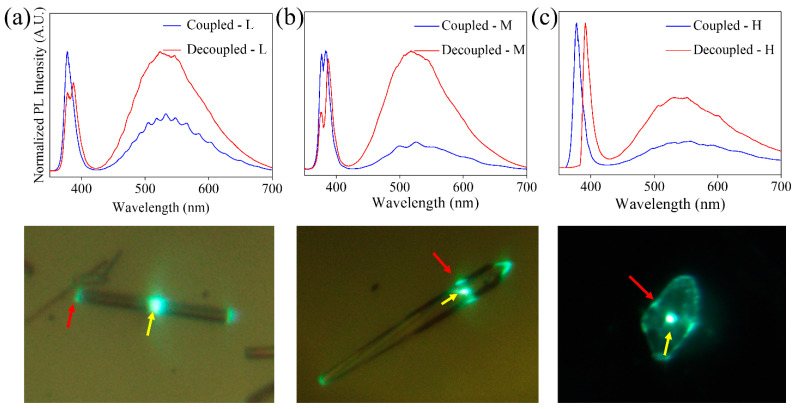
Analysis by photoluminescence spectra of light guidance on (**a**) L, (**b**) M and (**c**) H. Below each of the spectra, the optical image of the structure where the measurements were made is shown. The yellow arrows indicate the excitation point, while the red arrows indicate where the light is collected for the decoupled spectra.

The experimental system used allows us to analyze the guided emission by decoupling the excitation beam from the light collection point. In this way, luminescence can be excited at one point and collected at another one, enabling the comparison of spectra collected at the point of excitation and at the exit point of the guided light. Figure 11 shows the spectra obtained on structures of L-M-H samples. The blue line corresponds to the spectra recorded by exciting and collecting the luminescence on the same point (yellow arrow in the optical image), while in the spectra indicated by the red line, the beam has been decoupled. Excitation takes place in the region indicated by the yellow arrow, and the guided light is collected in the position marked by the red arrow. In general, two peaks are observed in the UV region due to band–band transition and shallow defects. When decoupling the beam, the ratio between the intensity of both peaks varies, showing a displacement at higher wavelengths. This is due to the self-absorption of the luminescence arising from the band to band transition, which is efficient in that spectral region. These changes are frequently observed in ZnO elongated structures [44,45].

Some of the spectra shown in Figure 11 show clear modulations (Figure 11a), whose presence has been explained as a result of an interference phenomenon when the light is confined inside the structure. Due to the high refractive index of ZnO (n_ZnO_ ≈ 2) and the cross-section geometry of the structures, the light can be confined by internal reflections. The light beams travel the same optical path within the cavity, producing interference between successive beams. The observed modulations confirm the possibility of confining light in the structures of the ZnO:K system analyzed. A detailed analysis of one of these cavities has been carried out based on previous results on ZnO:Li needle-like structures grown by the same method [30]. This makes it possible to select a structure that meets the conditions required to obtain a better confinement of light: hexagonal geometry, constant size of the cross-section and size of approximately 1 μm in diameter. In these conditions, a detailed study of the available resonant modes and calculations of refractive index, quality factor and fineness is possible [30].

**Figure 12:**
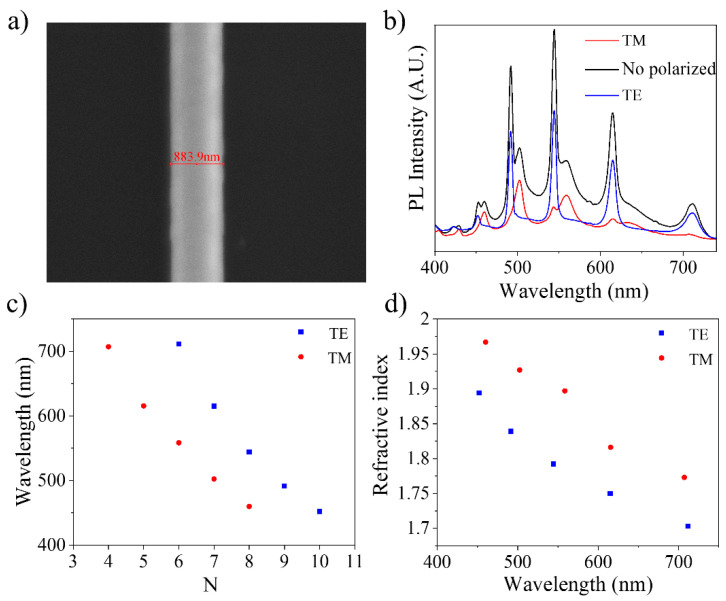
Analysis of structures as resonant cavities in ZnO:K. (**a**) Electron micrograph of the hexagonal structure with a width of 883.9 nm. (**b**) Photoluminescence spectrum with the total component and polarized components. (**c**) Calculation of the interference order and (**d**) the refractive index as a function of wavelength.

For the sake of illustration, we selected a typical needle-like structure, with constant section on which the photoluminescence spectrum has been taken in both polarizations of the emitted light (Figure 12a,b). The results for the interference order (N) and the refractive index (n) are shown in Figure 12c,d. The values for N and n were calculated through the geometrical optical path of the light inside the cavity (δ_op_) using the following equations for both polarizations, as reported in [30].

(1) $$\lambda_{\mathrm{TM}}=\frac{n\cdot \delta_{\mathrm{op}}}{N+\frac{6}{\pi }\cdot \arctan\left(\frac{1}{n}\sqrt{3n^{2}-4}\right)}$$

(2) $$\lambda_{\mathrm{TE}}=\frac{n\cdot \delta_{\mathrm{op}}}{\left(N-3\right)+\frac{6}{\pi }\cdot \arctan\left(n\cdot \sqrt{3n^{2}-4}\right)}$$

The refractive index obtained is below the values expected by the Sellmeier dispersion equation for pure zinc oxide [46]. In turn, the value of n also presents a decrease compared to the results obtained in previous works for structures doped with lithium [30]. However, the works of Baizid et al. [47] and Shanmuganathan et al. [5] show that potassium doping increases the refractive index with respect to undoped ZnO. Figure 12 also shows a large separation between the calculated values for each of the polarizations. Although this separation can be attributed to a poor fit of the mathematical model used to perform the calculation, this deviation may also be due to a poorer quality of the optical cavity. Khanum et al. [25] studied the formation of resonant cavities in microspheres of ZnO:Li and ZnO:Na. Their studies point out that the best results are obtained when Li is used as a dopant, but in quantities greater than 2 at %, Li degrades the crystallinity of ZnO, affecting the behavior of cavities. The same happens when doping with Na. An analogous behavior can be expected to occur in potassium. Due to the size of the K ion, practically double that of Zn, and the different positions it can adopt within the ZnO lattice (K_i_ or K_Zn_), high distortions are introduced, which end up degrading the behavior as a resonant optical cavity.

Quality factor (Q) and fineness (F) of this particular cavity were calculated and are shown in Table 3. Although the quality of the optical cavity is not as high as that of Li-doped ZnO structures [30], the results show adequate values for both parameters. For a wavelength of 544 nm, where the best result is found, the calculated quality factor is 80.68, indicating that these structures could be used in future photonics applications.

## 4. Conclusions

The growth of micro- and nanostructures of K-doped ZnO was achieved by the Vapor–Solid method. The main morphologies obtained were elongated structures, but the formation of ribbons and triangular structures was also detected. The results indicate that the K ions can be introduced in different positions in the ZnO lattice depending on the amount of potassium in the sample. Firstly, where the K concentration is low (L sample), potassium is incorporated in interstitial positions, whereas substitutional positions of Zn are occupied when the amount of K is increased (M-H sample). The formation of ribbons and triangular structures is the morphological consequence of the lattice distortions introduced by the K_i_ since this position favors growth in the (0001) plane. The crystal orientation of these structures was confirmed by EBSD measurements. Cathodoluminescence results show variations in the relative intensities of the emission bands as the percentage of K in the initial mixture of precursors increases, revealing the influence of a higher concentration of defects. This fact is also reflected in the Raman spectra, from which the low crystalline quality in the heavily doped samples is evident.

On the other hand, structures with a second composition were detected. The EDX and Raman spectroscopy analysis led to the formation of potassium sulfate. The morphology is quite distinguishable due to its ball shape. It has been observed that when the K amount exceeds the solubility threshold in K-doped ZnO structures, the dopant migrates to the surface and reacts with the sulfur in the atmosphere from the ZnS precursor, covering the structures with a K_2_SO_4_ film.

Finally, the PL study shows that the micro- and nanostructures of K-doped ZnO can act as waveguides and resonant cavities forming Whispering Gallery Modes. Quality factor and fineness were calculated for different wavelengths; in particular, for 544 nm, the values obtained were suitable, opening the door to real applications in optoelectronics.

## Figures and Tables

**Table 1:** Calculation of the FWHM of the peaks at 100 and 437 cm^−1^ for each sample.

	E_2_^low^ Mode		E_2_^high^ Mode	
	Position (cm^−1^)	FWHM	Position (cm^−1^)	FWHM
ZnO undoped	100	8.16	439	7.46
L	98	5.62	437	7.96
M	97	11.65	437	10.94
H	97	9.22	437	10.84

**Table 2:** Raman modes detected in the spectra on the coated triangles. The values are consistent with those reported in [37] for K_2_SO_4_.

Position (cm^−1^)	Symmetry
452	ν_2_ (E)
619	ν_4_ (F_2_)
626	ν_4_ (F_2_)
982	ν_1_ (A_1_)
1090	ν_3_ (F_2_)
1103	ν_3_ (F_2_)
1145	ν_3_ (F_2_)

**Table 3:** Calculation of the quality factor (Q) and fineness (F) of the ZnO:K structure studied as a resonant cavity.

Wavelength (nm)	Quality Factor (Q)	Fineness (F)
454.05	61.39	5.36
491.54	75.62	8.08
544.08	80.68	10.46
614.64	61.29	9.50

## Data Availability

The data presented in this study are available on request from the corresponding author. The data are not publicly available due to technical reasons.
